# Influence of habitat management and selected environmental parameters on the ground-living communities of harvestmen (Opiliones) in the historical park in Rusovce (Slovakia)

**DOI:** 10.1007/s10661-024-13233-6

**Published:** 2024-10-15

**Authors:** Juraj Litavský, Oto Majzlan, Vladimír Langraf, Hubert Žarnovičan

**Affiliations:** 1 Department of Environmental Ecology and Landscape Management, Faculty of Natural Sciences, Comenius University in Bratislava, Mlynská Dolina, Ilkovičova 6, 842 15 Bratislava, Slovakia; 2 Department of Zoology and Anthropology, Faculty of Natural Sciences and Informatics, Constantine the Philosopher University in Nitra, Trieda Andreja Hlinku 1, 949 74 Nitra, Slovakia

**Keywords:** Habitat management, Biodiversity, Bioindicators, Urban green space, Historical park, Opiliones

## Abstract

City parks serve as valuable refuges for invertebrates in the urban environment, which are constantly exposed to human influence caused by management practices in the parks. Many harvestman species are suitable bioindicators for monitoring ecological change; however, their effective use in environmental assessment necessitates to expand the insufficient knowledge of ecological environmental specificities of their communities. We studied the diversity and dynamics of harvestman (Opiliones) communities in the historical park in Rusovce, situated in the southwestern part of Bratislava (Slovakia). Seven study sites were investigated, representing areas subjected to different management practices. The research was conducted from March 2019 to April 2020, using pitfall traps. We evaluated the impact of microclimatic variables (soil and air temperature), environmental characteristics (plant diversity and cover of vegetation layers, age of forest stands, thickness of the litter layer), and management practices on the structure of harvestman communities. The study revealed the response of specific harvestman species to temperature variations, emphasizing the importance of microclimatic conditions. Environmental variables, such as the richness of plant species in the shrub layer, the cover of the herb and shrub vegetation layers, and the age of the forest stands, were identified as key factors that influence the dispersal of harvestman species. Furthermore, management practices, especially the presence of monoculture tree plantations, significantly affected the species composition of harvestmen. Examining the sensitivity of Opiliones, important bioindicators, to these factors is crucial for implementing effective conservation strategies in urban green spaces and anthropogenically influenced ecosystems.

## Introduction

Harvestmen are arachnids that play a vital role in terrestrial ecosystems. Their presence and diversity are often indicative of the ecological health of a given habitat (Litavský et al., 2019a, b; Menta & Remelli, 2020; Pinto-da-Rocha et al., 2007; Tourinho et al., 2020). Understanding the factors influencing harvestman communities is crucial for effective biodiversity conservation and habitat management (Drummond et al., 2010; Muster et al., 2014; Stašiov et al., 2011, 2021). The high habitat requirements of many species of harvestmen are expressed by a high affinity for the different types of habitats (Klimeš, 1997), structures (Bragagnolo et al. (2007); Curtis & Machado, 2007; Spitzer et al., 2008), and specific microclimatic conditions (Andrade et al., 2022; Mitov & Stoyanov, 2005) and thus with a very strong sensitivity to environmental changes (Litavský et al., 2019a). Given their sensitivity to habitat changes, harvestmen serve as valuable bioindicators for monitoring biodiversity loss, particularly in response to anthropogenic impacts. Due to such a narrow ecological valence, many authors consider these organisms to be very suitable bioindicators and monitoring organisms in the assessment and planning of nature conservation (Gerlach et al., 2013; Holzinger, 2010; Klimeš, 1997; Komposch, 1997, 2006, 2009; Litavský et al., 2019a; Majzlan & Hazuchová, 1997; Merino-Sáinz & Anadón, 2015; Pinto-da-Rocha et al., 2007; Stašiov, 2001; Vasconcellos et al., 2013).

Biodiversity is rapidly declining at the global level. Urbanization (Peng et al., 2020; Sala et al., 2000; Seto et al., 2012), the development of intensive agriculture (Raven & Wagner, 2021; Tsiafouli et al., 2014), economic growth (Otero et al., 2020), and infrastructure development (Laurance et al., 2015) have a significant impact on the global decline of biodiversity. For this reason, urban green spaces such as historical parks play a very important role in supporting biodiversity in cities (Aronson et al., 2014; Lepczyk et al., 2017). Therefore, it is currently necessary to put great emphasis on the importance of urban green spaces for the support and conservation of biodiversity in cities. Such areas include, for example, botanical, zoological, and private gardens, alleys, cemeteries, recreational areas, and, above all, parks, such as the historical park in Rusovce in Bratislava (Slovakia).

Regarding the history of the study area in terms of its land use, historical maps indicate that in the second half of the eighteenth century, a baroque garden was established here (Reháčková, 2011). According to this author, in 1856, the regularly arranged baroque garden was transformed into the then-modern style of naturalistic landscape parks. Thus, a large landscape park was created, incorporating the modified meander of the Danube — Rusovské rameno. This watercourse divided the park into two parts: the part south of the manor house, where the original baroque garden once stood, extending to the Rusovské rameno meander, and the part beyond the meander, spreading north-eastward toward the fields. According to Vágenknechtová (1992), the final developmental phase of the park’s landscaping, characterized by typical features of the late period of English landscape parks, dates back to the end of the nineteenth century. After the Second World War, the park in Rusovce was nationalized, and the Slovak Folk Art Ensemble became the owner. However, the unmaintained mansion gradually deteriorated over time, leading to plans for a comprehensive reconstruction of the entire area (Reháčková, 2009).

From 2019 to 2020, south of the mansion to the oxbow lake Rusovské rameno, park revitalization took place through arboricultural interventions (cutting down some trees, pruning branches, removing invasive plants, mowing). We managed to record these management impacts on harvestman communities, which were implemented directly at our study sites (S1–S3) during our 1-year research, and compare them with four other sites (S4–S7) on the opposite side of the oxbow lake Rusovské rameno, which represent forest habitats where no active management has been carried out in the past 8 years.

The aim of our research is to observe the dynamics and determinants of harvestman (Opiliones) communities within the historical park in Rusovce. Using extensive field data and statistical analyses, the research aims to elucidate the impact of microclimatic variables (soil temperature in 6 cm depth, T1; air temperature at ground level + 2 cm, T2; and 15 cm above ground, T3), environmental characteristics (plant diversity and cover of vegetation layers, age of forest stands, thickness of the litter layer) and management practices (mowing, cutting trees, trampling of vegetation, the presence of invasive plant species, and the presence of monoculture tree plantations) on Opiliones spatial distribution and species composition. By investigating complex interactions, we try to find out valuable insights about the effects of environmental properties and management measures implemented in green spaces, such as the historical park in Rusovce, on these often-overlooked arachnids.

In addition to the objectives, we also set six hypotheses. We hypothesized that (1) higher soil temperatures (T1) will correlate positively with harvestman abundance, as warmer soil may enhance their activity and reproductive success, (2) air temperature at ground level (T2), and above ground (T3) will exhibit a positive relationship with the species richness, since warmer temperatures are generally favorable for ectothermic organisms such as harvestmen, (3) increased plant diversity and coverage of vegetation layers will lead to higher harvestman species richness and abundance, as these factors provide more microhabitats and resources, (4) the presence of invasive plant species will negatively impact native harvestman populations by altering habitat structure and reducing food availability, (5) areas dominated by monoculture tree plantations will have a lower harvestman diversity compared to mixed species forests, as monocultures typically provide less habitat complexity and fewer ecological niches, and (6) sites with active management (e.g., mowing, tree cutting, trampling) will show lower harvestman diversity and abundance compared to unmanaged sites, due to habitat disturbance and loss of microhabitats.

## Material and methods

### Study sites

**Fig. 1:**
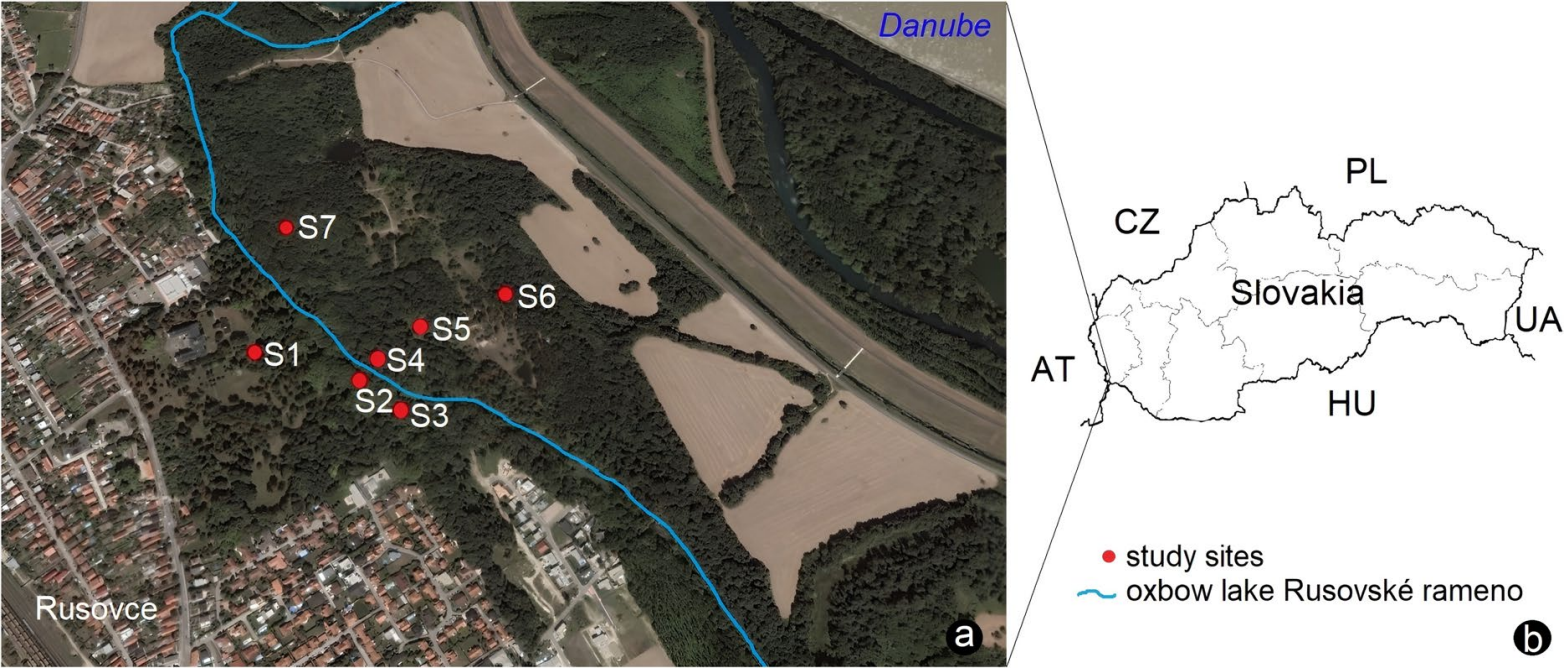
Illustration showing **a** the study area within the historical park in Rusovce (Bratislava, Slovakia) and **b** the location of the study area on the map of Slovakia: S1–S7, study sites; AT, Austria; CZ, Czech Republic; HU, Hungary; PL, Poland; UA, Ukraine

The research of harvestmen (Opiliones) was carried out at seven study sites located in the historical park of Rusovce, located in the southwestern part of the city of Bratislava. The study sites are placed approximately 1–1.4 km away from the Danube River (Fig. 1). Currently, only the area in front of the oxbow lake Rusovské rameno is considered the park, covering an area of approximately 16 hectares. According to Reháčková (2011), in the past, a part behind the oxbow lake Rusovské rameno also belonged to the park, forming a cohesive unit, as evidenced by the species composition of the plants. Therefore, our research was carried out in both parts within the historical boundaries of the park area. The total area of the entire park is approximately 36 hectares and represents two types of areas with two management approaches, distinguishing between the park-maintained area where more intensive management measures are implemented (study sites S1–S3) and the area with less or no management interventions (sites S4–S7), where no active management has taken place in the last 8 years (cutting down trees, pruning branches, removing invasive plants, mowing), but here, there are two types of monoculture forests. These differently managed parts of the historical park are separated by the oxbow lake Rusovské rameno.

During the planning of the experiment and the field reconnaissance, our aim was to record all types of predominantly forest habitats present throughout the park area. The main criteria were the composition of the plants (woody species) and the way the area was managed. In total, we selected seven different types of habitats that are dominant within the historical park in Rusovce.

### Sampling

The research was conducted from 1 March 2019 to 1 April 2020. The harvestmen (Opiliones) were collected using pitfall traps (Krajčovičová et al., 2022), using 0.5-l plastic cups (9 cm opening diameter) filled one-third with a 4% formaldehyde fixative solution. Five traps were set in a row at each study site, with a minimum of 5 m separating each cup. The transects were positioned in the center of the monitored sites. Approximately every 2 weeks, the traps were emptied and replenished. For each sampling period, the contents of five traps from each site and date were combined and treated as a composite sample. According to Martens (1978) and Krajčovičová et al. (2022), the obtained material was sorted in the laboratory and identified to the species level. The samples were fixed in 75% ethanol and deposited at the Faculty of Natural Sciences of the Comenius University in Bratislava. The scientific nomenclature was used according to the work of Kury et al. (2023), which currently represents the best-compiled World Catalogue of Opiliones.

Regarding management measures, during our research, within individual study sites, we documented activities such as mowing, cutting trees, and trampling of vegetation, as well as the condition of habitats altered by human intervention in the environment: the presence of invasive plant species and the presence of monoculture forests (Table 1).

**Table 1:** Documented activities and habitat conditions caused by ongoing management measures in the monitored area

Management	S1	S2	S3	S4	S5	S6	S7
Trampling vegetation	x						
Invasive species	x	x			x		
Cutting trees			x		x		
Mowing	x						
Monoculture						x	x

We conducted estimations of the cover of tree, shrub, and herb layers at the study sites, and sampled species occurring in individual layers, all in a sampling area of 300 m^2^ (30 m × 10 m). For each plant species, we assigned indices (1–3) based on Tansley and Chipp (1926): 1 denoting less than 1% dominance, 2 denoting 1 to 50% dominance, and 3 denoting more than 50% dominance. The nomenclature of the vascular plants was interpreted following the Govaerts (2024). The age of the trees at the study sites (Age [year]) was determined based on historical maps and estimates based on our own professional experience. During our fieldwork, we measured the average depth of the litter layer (Litter [cm]).

Microclimatic data were recorded every 15 min from 1 March 2019 to 1 April 2020 using TMS-4 loggers (Wild et al., 2019) located in the center of the study site. We had 7 data loggers in total. TMS-4 loggers measure soil temperature in 6 cm depth (T1), air temperature at ground level + 2 cm (T2) and 15 cm above ground (T3). Furthermore, soil moisture is measured up to 11 cm depth, but we did not evaluate the recorded soil moisture values because some moisture sensors did not register moisture at certain times. Therefore, we wanted to avoid an incorrect interpretation of the data.

### Statistical analyses

In redundancy analysis (RDA), we expressed the relationship between the research variables (temperature (T1–T3), management (invasive species, trampling vegetation, mowing, cutting trees, monoculture tree plantations), E_1_–E_3_ [%], E_1_–E_3_ species, age, litter) and spatial dispersion of Opiliones species. Thus, with RDA analysis, we tested the spatial dispersion of Opiliones under the influence of environmental variables in 3 separate analyses. The first aims to assess the impact of temperature (T1–T3), the second aims to assess the impact of management (invasive species, trampling vegetation, mowing, cutting trees, monoculture tree plantations), and the third aims to assess the impact of E_1_–E_3_ [%], E_1_–E_3_ species, age, litter. RDA analysis was used in all 3 separate analyses, because length of gradient (SD) = 1.1 (length of gradient is part of the RDA model). Based on this value, linear models must be chosen for analyses. Analyses continued according to the default settings of the Canoco5 software. We tested the statistical significance of the variables using the Monte Carlo permutation test in (iteration 499) the Canoco5 program (Ter Braak & Šmilauer, 2012). We followed Losos et al. (1984) in using the dominance classes: > 10%, eudominant; 5–10%, dominant; 2–5%, subdominant; 1–2%, recedent; less than 1%, subrecedent.

We performed the analysis of rarefaction curves in the program R version 4.1.3 (R Core Team, 2020). Rarefaction curves were used to predict the occurrence of species richness in study areas (1–7). We performed the analysis in program R version 4.1.3 (R Core Team, 2020).

## Results

Based on the conducted estimations of the cover of tree, shrub, and herb layers, we characterized the study sites (S1–S7) as follows:

- S1 (48°03ʹ08.40ʺ N, 17°09ʹ09.50ʺ E, 134 m a.s.l.) — Represents a shrub-meadow ecotone influenced by mowing, trampling of vegetation, and the presence of invasive plant species. The tree layer (E_3_) is absent. The shrub layer (E_2_) is represented by one species — *Taxus baccata* 2 with a total coverage of 30%. The herbaceous layer (E_1_ species) is represented by 42 plant taxa with a total coverage of 100%
- S2 (48°03ʹ05.50ʺ N, 17°09ʹ19.50ʺ E, 132 m a.s.l.) — Plane tree alley, with *Platanus* × *hispanica* Mill. ex Münchh. 3 in the tree layer with a total coverage of 90%. The shrub layer (E_2_) is represented by *Acer platanoides* 2, *A*. *pseudoplatanus* 2, *Fraxinus excelsior* 2, *Sambucus nigra* 2, *Acer campestre* 1, *Prunus avium* 1, *Prunus padus* 1, *Platanus* × *hispanica* Mill. ex Münchh. 1 and *Prunus spinosa* 1 with a total coverage of 75%. The herbaceous layer includes 14 plant species with a total coverage of 20%
- S3 (48°03ʹ04.0ʺ N, 17°09ʹ22.70ʺ E, 152 m a.s.l.) — A hardwood floodplain forest with *Acer pseudoplatanus* 2, *Fraxinus excelsior* 2, *Quercus robur* 2, and *Ulmus glabra* 2 in the tree layer, with a total coverage of 70%. In the shrub layer (E_2_), the following plant species are represented: *Acer campestre* 2, *A*. *pseudoplatanus* 2, and *Prunus padus* 2, with a total coverage of 15%. The herbaceous layer (E_1_) is represented by 15 plant species with a total coverage of 95%
- S4 (48°03ʹ07.0ʺ N, 17°09ʹ20.30ʺ E, 150 m a.s.l.) –– A hardwood forest in the floodplain, situated near the oxbow lake of Rusovské Rameno, with *Fraxinus excelsior* 3 and *Populus alba* 2 in the tree layer with a total coverage of 55%. The shrub layer (E_2_) is represented by *Acer pseudoplatanus* 3, *A*. *campestre* 2, *A*. *platanoides* 2, *Euonymus europaeus* 2, *Crataegus monogyna* 2, *Cornus sanguinea* 2, *Berberis vulgaris* 1, *Prunus avium* 1, *Carpinus betulus* 1, *Prunus padus* 1, *Rosa canina* agg. 1, and *Ulmus glabra*, with a total coverage of 60%. The herbaceous layer consists of 20 plant taxa with a coverage of 70%
- S5 (48°03ʹ09.70ʺ N, 17°09ʹ23.70ʺ E, 127 m a.s.l.) — Represents a semi-open, young stand with *Betula pendula* 2, *Populus alba* 2*, Prunus domestica* subsp. *insititia* 2 and *Juglans regia* 1 in the tree layer, with a coverage of 45%. The shrub layer is represented by *Acer pseudoplatanus* 2, *Betula pendula* 2, *Fraxinus excelsior* 2, *Pinus sylvestris* 2, *Salix cinerea* 2, *Tilia* × *europaea* 2, and *Carpinus betulus* 1, with a total c overage of 60%. The herbaceous layer is represented by 28 plant taxa with a total coverage of 95%
- S6 (48°03ʹ11.80ʺ N, 17°09ʹ31.10ʺ E, 130 m a.s.l.) — Represents a 50-year-old forest stand with *Pinus nigra* 3 and *Tilia* × *europaea* 2 in the tree layer and a coverage of 90%. The shrub layer consists of the species: *Crataegus monogyna* 2, *Tilia* × *europaea* 2, and *Cotinus coggygria* 1 with a coverage of 4%. The herbaceous layer consists of 13 plant species with a coverage of 8%
- S7 (48°03ʹ16.10ʺ N, 17°09ʹ11.10ʺ E, 158 m a.s.l.) — A 20-year-old forest stand with *Tilia* × *europaea* 3 and *Acer campestre* 1 in the tree layer with a total coverage of 95%. The shrub layer includes *Crataegus laevigata* 2, *C*. *monogyna* 2, *Lonicera xylosteum* 2, *Acer campestre* 1, *A*. *pseudoplatanus* 1, *Frangula alnus* 1, *Ligustrum vulgare* 1, *Taxus baccata* 1, and *Tilia* × *europaea* 1 with a coverage of 20%. The herbaceous layer is represented by 22 plant species with a total coverage of 75%

Concerning environmental characteristics, we opted to observe the parameters that could influence the composition of harvestman communities (Table 2).

**Table 2:** The analyzed environmental parameters: age, litter depth, stand canopy of individual layers (E_1_, E_2_, E_3_ [%]), the number of plant species of individual layers (E_1_, E_2_, E_3_ plant species) and the study sites S1–S7

Environmental parameters	S1	S2	S3	S4	S5	S6	S7
Age (year)	20	120	70	70	8	50	20
Litter depth (cm)	1.5	7	3.1	3	1.8	3.4	2.5
E_1_ (%)	100	20	95	70	95	8	75
E_2_ (%)	30	75	15	60	60	4	20
E_3_ (%)	0	90	70	55	45	90	95
E_1_ species	42	14	15	20	28	13	22
E_2_ species	1	9	3	12	7	3	9
E_3_ species	0	1	4	2	4	2	2

During our investigation, we recorded a total of 2291 individuals belonging to 13 species and 4 families at the investigated locations. We confirmed the eudominant representation in the species *Nemastoma bidentatum sparsum* (36.71%), *Nelima sempronii* (22.48%), and *Oligolophus tridens* (18.51%) (Table 3). At all study sites located in the historical park in Rusovce, we recorded *Trogulus tricarinatus*, *Nemastoma bidentatum sparsum*, *Mitostoma chrysomelas*, *Astrobunus laevipes*, and *Nelima sempronii*. The least numerous species were *Opilio saxatilis*, *Lophopilio palpinalis*, and *Opilio canestrinii*. The species that were captured at only one site for the entire research period were *Lophopilio palpinalis* (S3) and *Opilio saxatilis* (S7). Regarding the number of recorded individuals of harvestmen, the highest abundance was observed at study sites S3 and S4 across all study areas. These sites are located near the oxbow lake Rusovské rameno and represent the original, least human-affected habitats of hardwood floodplain forests, as evidenced by their composition of plant species. The difference is that site S3 is in the park area where tree thinning and branch pruning occurred in the early months of our study. This management measure clearly did not negatively affect the harvestmen communities, as was also statistically proven.

**Table 3:** Recorded species of harvestmen at the study sites (S1–S7)

Familia/species	Code	S1	S2	S3	S4	S5	S6	S7	∑ *N*	∑ %
Nemastomatidae Simon, 1872		104	165	410	98	48	14	46	885	38.63%
*Mitostoma chrysomelas* (Hermann, 804)	MitsChrs	1	2	13	17	7	1	3	44	1.92%
*Nemastoma bidentatum sparsum* (Gruber & Martens, 1968)	NemBidSp	103	163	397	81	41	13	43	841	36.71%
Phalangiidae Latreille, 1802		1	24	167	229	21	62	143	647	28.24%
*Lacinius dentiger* (Koch, 1847)	LacnDent	0	0	1	4	0	57	3	65	2.84%
*Lacinius ephippiatus* (Koch, 1835)	LacnEphp	0	0	6	14	6	0	36	62	2.71%
*Lophopilio palpinalis* (Herbst, 1799)	LophPalp	0	0	2	0	0	0	0	2	0.09%
*Oligolophus tridens* (Koch, 1836)	OligTrid	1	23	154	205	11	0	30	424	18.51%
*Opilio canestrinii* (Thorell, 1876)	OpilCans	0	1	1	0	4	1	0	7	0.31%
*Opilio saxatilis* Koch, 1839	OpilSax	0	0	0	0	0	0	1	1	0.04%
*Rilaena triangularis* (Herbst, 1799)	RilaTria	0	0	3	6	0	4	73	86	3.75%
Sclerosomatidae Simon, 1879		76	49	95	185	117	82	90	694	30.29%
*Astrobunus laevipes* (Canestrini, 1872)	AstrLaev	9	17	62	22	24	2	19	155	6.77%
*Leiobunum rotundum* (Latreille, 1798)	LeioRotn	0	1	1	10	8	0	4	24	1.05%
*Nelima sempronii* Szalay, 1951	NelmSemp	67	31	32	153	85	80	67	515	22.48%
Trogulidae Sundevall, 1833		8	5	14	12	5	16	5	65	2.84%
*Trogulus tricarinatus* (Linnaeus, 1767)	TrogTric	8	5	14	12	5	16	5	65	2.84%
∑		189	243	686	524	191	174	284	2291	100.00%

**Fig. 2:**
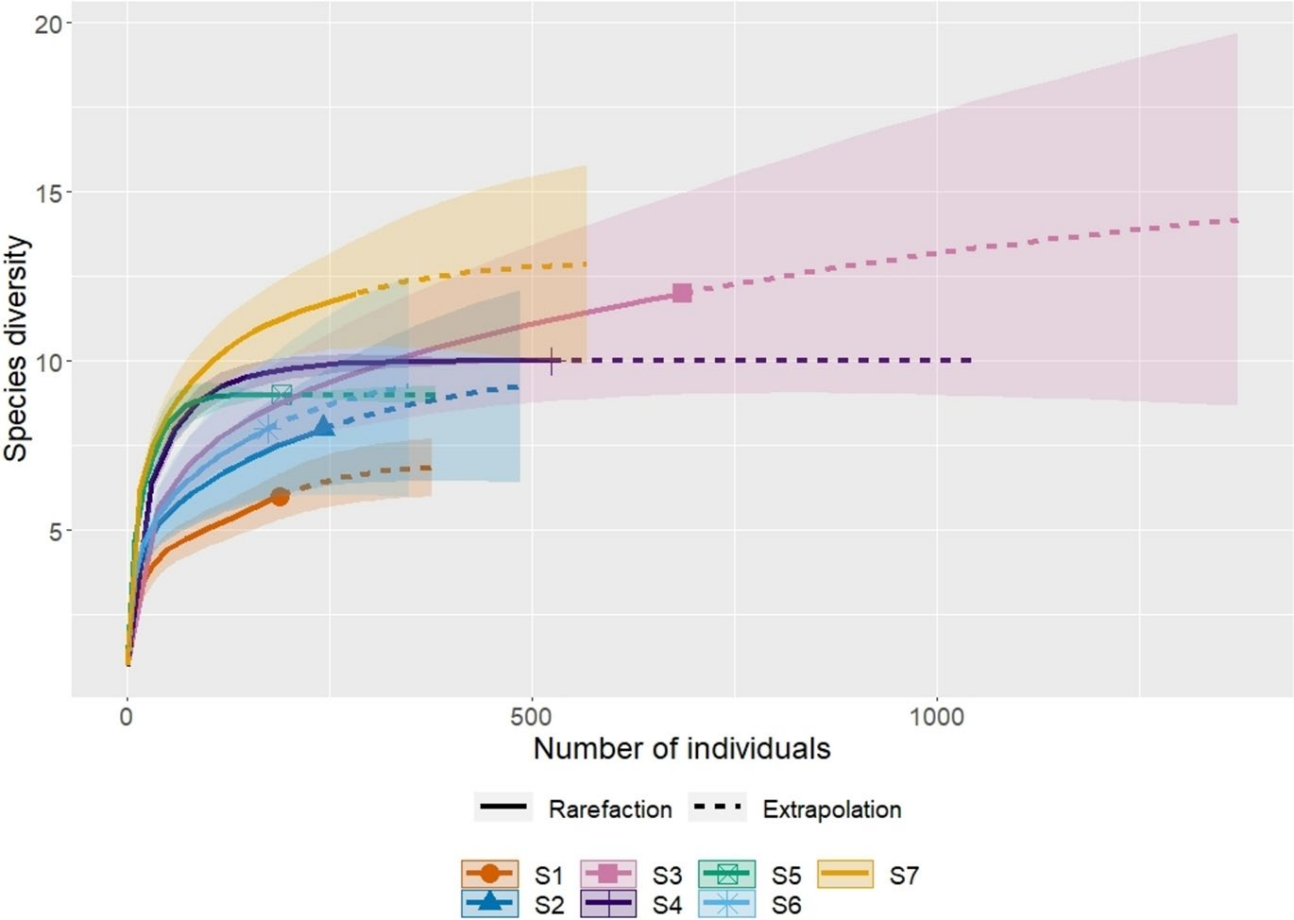
Prediction rarefaction curves of study areas (S1–S7)

With rarefaction curves, we confirmed the greatest species diversity at S3, then it decreased in the direction of S7, S2, and S6, S4, S5, and S1. With the same number of individuals, the order of species diversity from highest to lowest changed as follows: S7, S4, S6, S3, S5, S2, S1. Extrapolation to the highest number of individuals is estimated at S3 and subsequently decreases in the direction of S4, S7, S2, S5, and S1, S6. Thus, the prediction estimate also reflects the current state of abundance of individual study areas. The prediction estimate of species is at S3 = 18 species (currently 12 species), S7 = 15 species (currently 11 species), S6 = 12 species (currently 9 species), S2 = 12 species (currently 8 species), S4 = 10 species (currently 10 species), S5 = 9 species (currently 9 species), and S1 = 7 species (currently 6 species) (Fig. 2). Based on the analysis, we can establish a potential increase in species richness by 6 more species at S3, by 4 species at S7 and S2, by 3 species at S6, and by 1 species at S1.

Redundancy analysis (RDA, SD (length of gradient) = 1.1 on the first ordination axis) confirmed the significant influence of temperatures measured at different points (T1, soil temperature − 6 cm; T2, surface temperature + 2 cm; T3, air temperature + 15 cm) on the spatial structure of harvestman species. The results of significance for individual temperature points were as follows: T1 (*p* = 0.02, df = 2, *F* = 4.2), T2 (*p* = 0.04, df = 2, *F* = 3.8), T3 (*p* = 0.036, df = 2, *F* = 2.8). Spatial analysis explained 45.76% of the variability on the first axis and 62.73% on the second cumulative one. After adding the effect of temperature measured at different points, the variability on the first ordination axis increased to 66.96% and on the second cumulative axis to 78.81%.

**Fig. 3:**
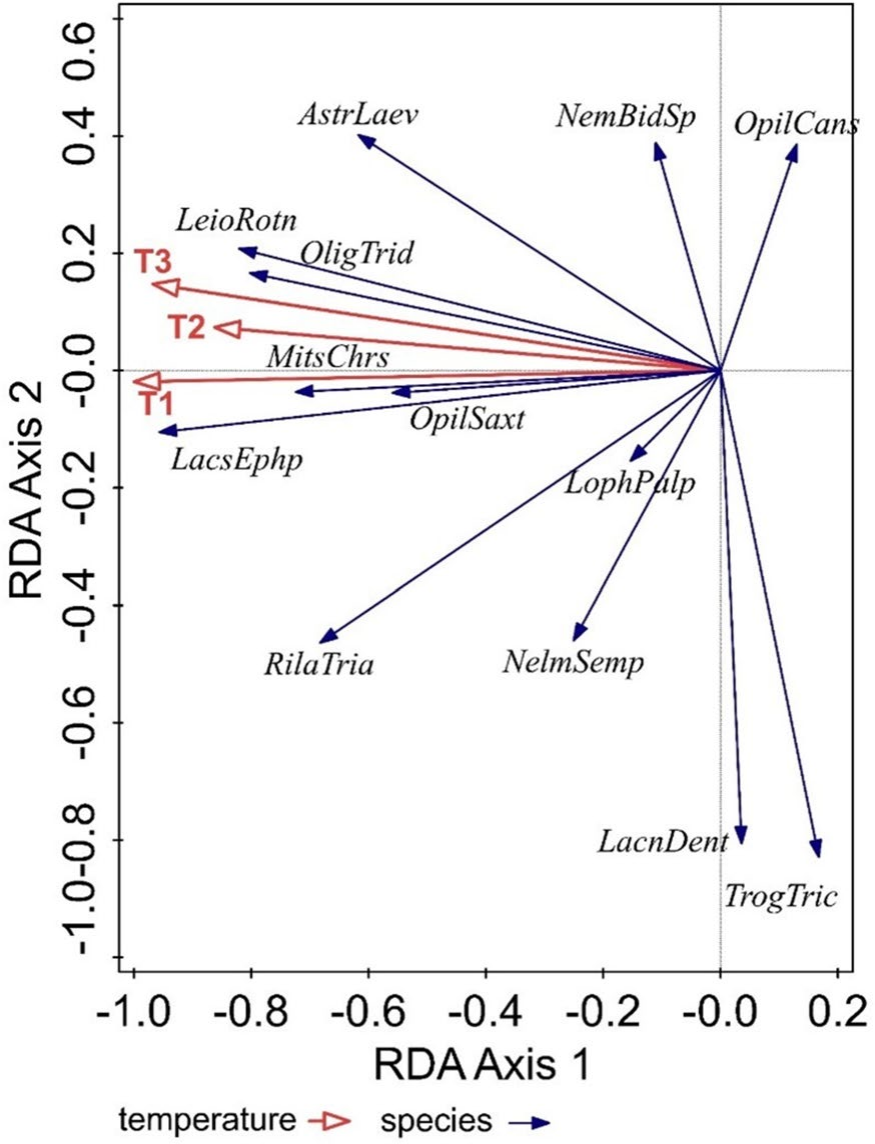
RDA analysis of the effect of temperature on the spatial structure of Opiliones species: *MitsChrs*, *Mitostoma chrysomelas*; *NemBidSp*, *Nemastoma bidentatum sparsum*; *LacnDent*, *Lacinius dentiger*; *LacnEphp*, *Lacinius ephippiatus*; *LophPalp*, *Lophopilio palpinalis*; *OligTrid*, *Oligolophus tridens*; *OpilCans*, *Opilio canestrinii*; *OpilSax*, *Opilio saxatilis*; *RilaTria*, *Rilaena triangularis*; *AstrLaev*, *Astrobunus laevipes*; *LeioRotn*, *Leiobunum rotundum*; *NelmSemp*, *Nelima sempronii*; *TrogTric*, *Trogulus tricarinatus*

In the ordination graph, we can see that the temperatures measured at various points T1–T3 had the greatest influence on the species *Leiobunum rotundum*, *Oligolophus tridens*, *Mitostoma chrysomelas*, *Opilio saxatilis*, and *Lacinius ephippiatus*. These species of harvestmen can be considered significant bioindicators that are sensitive to changes in environmental temperature. The other species were not affected by the temperature measured at different points (Fig. 3).

The influence of environmental variables (E_1_–E_3_ [%], E_1_–E_3_ species, age, litter) on the dispersion of species of the order of Opiliones was determined by RDA analyses (SD (length of gradient) = 1.1 on the first ordination axis). Spatial analysis explained 45.76% of the variability on the first axis and 62.73% on the second cumulative one. After adding the effect of environmental variables, the variability on the first ordination axis increased to 51.22% and on the second cumulative axis to 79.96%. We confirmed a statistically significant influence on the dispersion of species of Opiliones for the factors: the number of plant species in shrub layer, E_2_ species (*p* = 0.025, df = 7, *F* = 1.4); stand canopy of shrub vegetation layer, E_2_ [%] (*p* = 0.011, df = 7, F = 1.9); stand canopy of herb vegetation layer, E_1_ [%] (*p* = 0.023, df = 7, *F* = 2); and age of forest stands, age (*p* = 0.010, df = 7, *F* = 3.3). For other variables, we did not confirm a significant influence of E_1_ species (*p* = 0.826, df = 7, *F* = 1.2), E_3_ species (*p* = 0.558, df = 7, *F* = 1.3), E_3_ [%] (*p* = 0.774, df = 7, *F* = 1.1), and litter (*p* = 0.89, df = 7, *F* = 1.2).

**Fig. 4:**
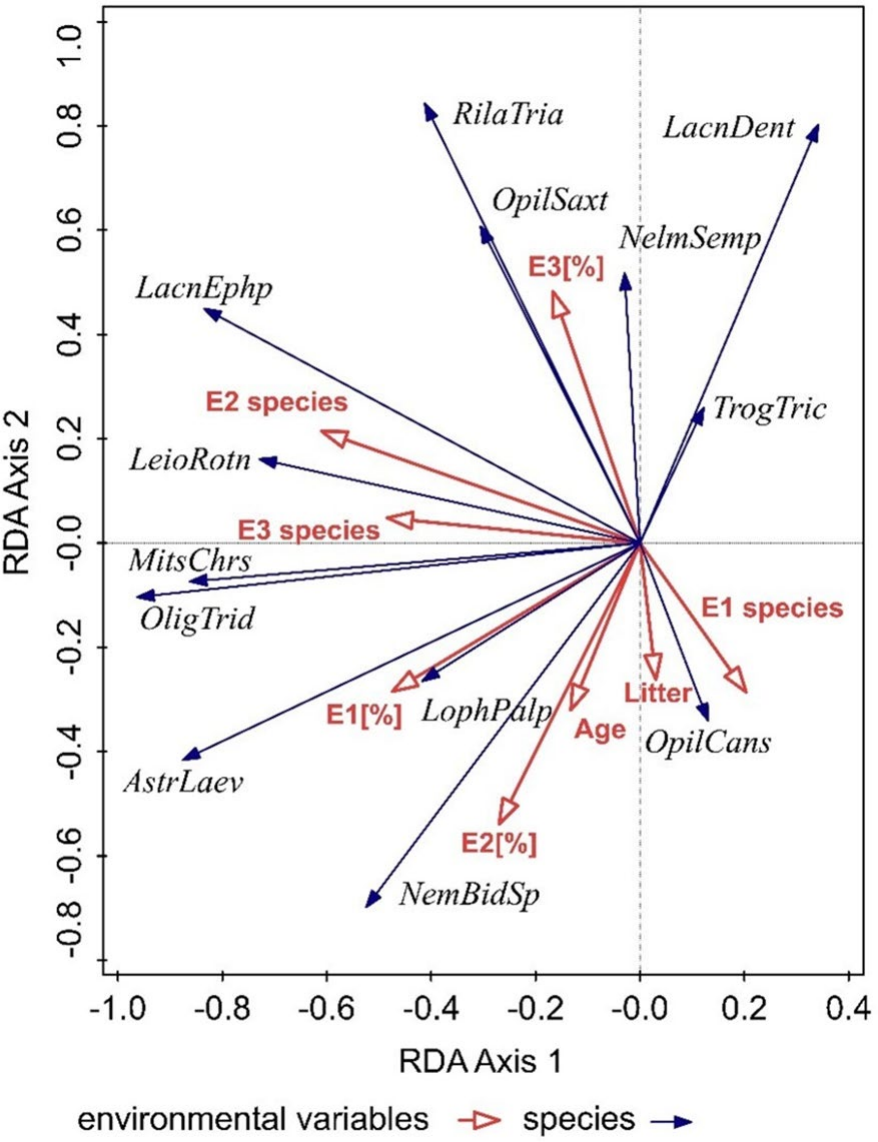
RDA analysis of the effect of environmental variables on the spatial structure of Opiliones species: *MitsChrs*, *Mitostoma chrysomelas*; *NemBidSp*, *Nemastoma bidentatum sparsum*; *LacnDent*, *Lacinius dentiger*; *LacnEphp*, *Lacinius ephippiatus*; *LophPalp*, *Lophopilio palpinalis*; *OligTrid*, *Oligolophus tridens*; *OpilCans*, *Opilio canestrinii*; *OpilSax*, *Opilio saxatilis*; *RilaTria*, *Rilaena triangularis*; *AstrLaev*, *Astrobunus laevipes*; *LeioRotn*, *Leiobunum rotundum*; *NelmSemp*, *Nelima sempronii*; *TrogTric*, *Trogulus tricarinatus*

From the results of the analysis, we confirmed that the stand canopy of the tree vegetation layer (E_3_ [%]) affects the species *Rilaena triangularis*, *Opilio saxatilis*, *Nelima sempronii*, *Lacinius dentiger*, and *Trogulus tricarinatus* the most. The plant species richness of shrub and tree vegetation layers (E_2_–E_3_ species) has the greatest influence on the species *Lacinius ephippiatus*, *Leiobunum rotundum*, *Mitostoma chrysomelas*, and *Oligolophus tridens*. The other variables such as the species richness of the herbaceous plant layer (E_1_ species), stand canopy of herbaceous vegetation layer (E_1_ [%]), stand canopy of shrub vegetation layer (E_2_ [%]), age of forest stands (age), and thickness of litter layer (litter) affected the occurrence of the species *Astrobunus laevipes*, *Lophopilio palpinalis*, *Nemastoma bidentatum sparsum*, and *Opilio canestrinii* (Fig. 4).

Using redundancy analysis (RDA, SD (length of gradient) = 1.1 on the first ordination axis), we determined the influence of management (invasive species, trampling vegetation, mowing, cutting trees, monoculture tree plantations) of the studied areas (S1–S7) on the spatial dispersion of Opiliones species. Spatial analysis explained 45.76% of the variability on the first axis and 62.73% on the second cumulative one. After adding the effect of management, the variability on the first ordination axis increased to 84.92% and on the second cumulative axis to 94.66%. Presence of monoculture tree plantations (*p* = 0.02, df = 3, *F* = 1.9) and invasion species (*p* = 0.015, df = 3, *F* = 1.7) had a statistically significant influence on the species of Opiliones composition. We did not confirm the significance of trampling vegetation (*p* = 0.55, df = 3, *F* = 1.5), mowing (*p* = 0.594, df = 3, *F* = 1.6), and cutting trees (*p* = 0.6, df = 3) for the other types of treatment.

Based on the results, we can see that the species *Nemastoma bidentatum sparsum*, *Astrobunus laevipes*, *Leiobunum rotundum*, *Lophopilio palpinalis*, *Mitostoma chrysomelas*, and *Oligolophus tridens* were most affected by the management of cutting trees.

**Fig. 5:**
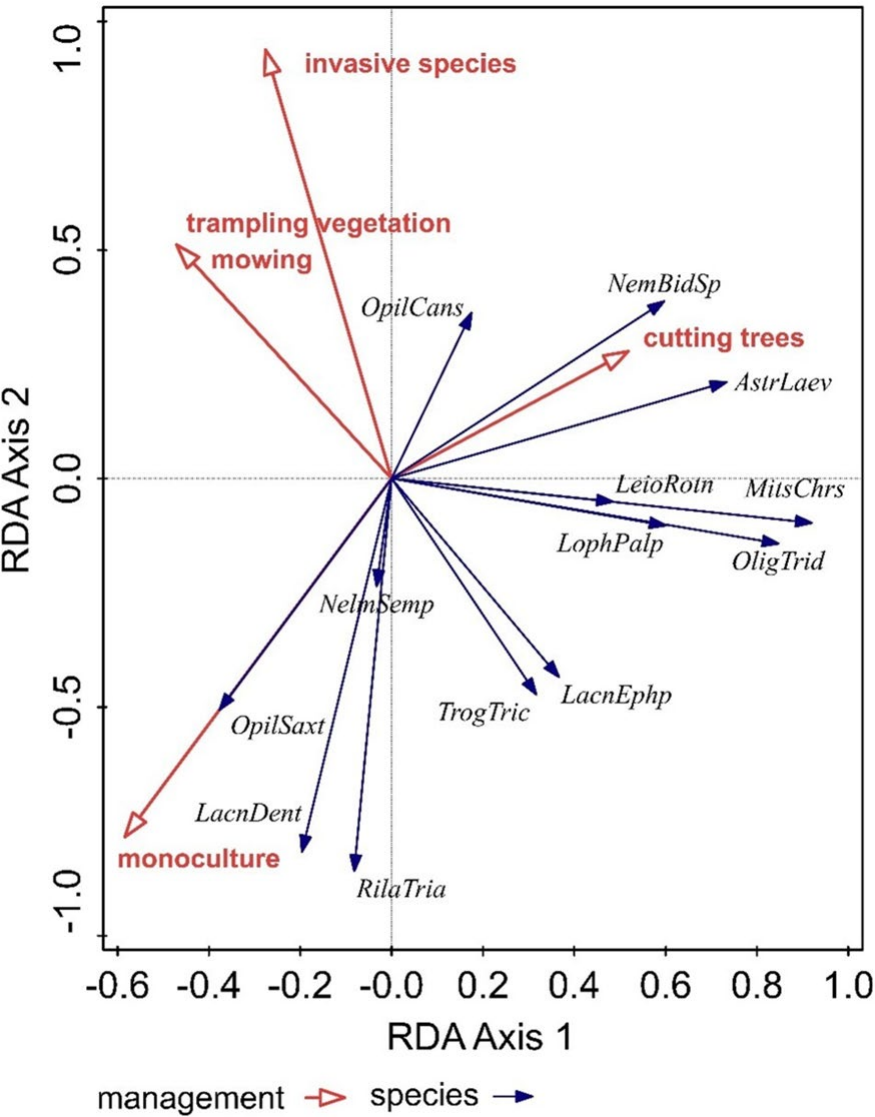
RDA analysis of the effect of management on the spatial structure of Opiliones species: *MitsChrs*, *Mitostoma chrysomelas*; *NemBidSp*, *Nemastoma bidentatum sparsum*; *LacnDent*, *Lacinius dentiger*; *LacnEphp*, *Lacinius ephippiatus*; *LophPalp*, *Lophopilio palpinalis*; *OligTrid*, *Oligolophus tridens*; *OpilCans*, *Opilio canestrinii*; *OpilSax*, *Opilio saxatilis*; *RilaTria*, *Rilaena triangularis*; *AstrLaev*, *Astrobunus laevipes*; *LeioRotn*, *Leiobunum rotundum*; *NelmSemp*, *Nelima sempronii*; *TrogTric*, *Trogulus tricarinatus*

Monoculture tree plantations positively affected the occurrence of the species *Lacinius dentiger*, *Lacinius ephippiatus*, *Opilio saxatilis*, *Rilaena triangularis*, and *Trogulus tricarinatus* (Fig. 5). These species of harvestmen can be considered important bioindicators preferring their occurrence in monoculture tree plantations within the lowlands. Especially *Lacinius dentiger* can be regarded as a typical indicator of planted pine monocultures.

## Discussion

The research of harvestmen (Opiliones) in urban green spaces reveals significant findings regarding species richness and abundance in various cities, particularly in relation to urban management practices and habitat characteristics. The results of our research revealed that even urban green spaces, such as those located in the historical park in Rusovce (Bratislava), could provide suitable conditions for harvestman communities with high abundance and species richness. The research identified 13 species, with species richness comparable to other urban areas. Notably, the proximity to the Danube River and its floodplain forests likely enhances habitat suitability, contributing to the observed species diversity. For comparison, Mitov and Stoyanov (2004) investigated harvestmen in the conditions of urban areas, who recorded 12 harvestman species in the city of Sofia, Bulgaria (*Carinostoma ornatum*, *Trogulus tricarinatus*, *Phalangium opilio*, *Opilio parietinus*, *O. saxatilis*, *O. dinaricus*, *O. ruzickai*, *Rilaena cf. serbica*, *Zachaeus crista*, *Lacinius horridus*, *L. dentiger*, and *Odiellus lendli*). Their findings suggest that urban green areas can serve as vital habitats for various species, despite the challenges posed by urbanization. Czechowski et al. (1981) conducted research on Warsaw’s harvestmen from 1974 to 1977, recording 13 species, with 12 species found in parks and other green elements (*Trogulus tricarinatus*, *Nemastoma lugubre*, *Leiobunum rupestre*, *L. blackwalli*, *Lacinius horridus*, *Lacinius ephippiatus*, *Rilaena triangularis*, *Lophopilio palpinalis*, *Opilio saxatalis*, *Nelima sempronii*, *Oligolophus tridens*, and *Phalangium opilio*), and only one species (*Opilio parietinus*) in the city center. Trigos-Peral et al. (2020) studied the opiliofauna in three categories of urban greenery in Warsaw (2 botanical gardens, 7 public parks, and 2 urban woodlands). The authors used pitfall trapping to capture epigeic invertebrates, placing 40 traps on each plot. In 2014, they recorded a total of 11 harvestman species in the entire study. Within public parks, similar to our sites S1–S3, they recorded a total of 9 species of harvestmen (minimum 2 and maximum 7 species/park). At similar study sites located in the park, we recorded a total of 13 harvestman species (at least 9 and up to 12 species per site). In urban woodlands, similar to our sites S4–S7, Trigos-Peral et al. (2020) captured a total of 7 species of harvestmen (4 species in one urban forest and 6 species in the second one). While, within the seven study sites representing urban woodlands, we recorded a total of 13 harvestman species (at least 8 and up to 11 species per site) (Table 3). The main reason for the lower number of recorded harvestman species in public parks and urban woodlands may be the fact that the traps were exposed for only 4 days (at the end of July and the beginning of August), making it impossible to capture the entire spectrum of species characteristic of the given locality.

In terms of the existing research on harvestmen (Opiliones) in Bratislava and its surroundings, several studies have been documented. The most significant works include those of Herman (1879), Daday (1918), Kratochvíl (1934), Bartoš (1939), Majzlan and Hazuchová (1997), and Litavský et al. (2019a). For comparison, the work of Bartoš (1939) can be partially used, as he recorded 15 species of harvestmen from Bratislava and its surroundings. These species include *Trogulus tricarinatus*, *Mitostoma chrysomelas*, *Nemastoma lugubre*, *Mitopus morio*, *Lacinius horridus*, *Lophopilio palpinalis*, *Phalangium opilio*, *Opilio parietinus*, *O. saxatilis*, *Egaenus convexus*, *Zachaeus crista*, *Rilaena triangularis*, *Astrobunus laevipes*, *Leiobunum rotundum*, and *L. rupestre* (probably *L. gracile*). Bartoš captured these species within the city, in gardens, under stones, and on tree trunks, as well as outside Bratislava, for example, in Marianka, Lozorno, and around the ruins of Pajštún Castle (at various elevations). Due to this fact, it is not possible to objectively compare the species composition of harvestmen recorded by Bartoš (1939) with our results, although nine identical species of Opiliones were recorded.

Given that the park in Rusovce is approximately 1 km away from the Danube River, and the studied area contains an oxbow lake of the Danube with floodplain forests, the most reliable work for comparing the obtained results on the species composition of harvestmen is the study by Litavský et al. (2019a). During the research of harvestmen in the floodplain forests of Bratislava from 2015 to 2016, the authors mentioned using the pitfall trapping method, recording 13 harvestmen species. The species that exhibited the highest dominance values throughout the years of the study were *Nemastoma bidentatum sparsum* (14.56%), *Egaenus convexus* (16.99%), *Astrobunus laevipes* (17.75%), and *Nelima sempronii* (23.12%). In addition to these, the authors also recorded the species *Trogulus tricarinatus*, *Nemastoma lugubre*, *Mitostoma chrysomelas*, *Lacinius ephippiatus*, *Opilio canestrinii*, *Phalangium opilio*, *Rilaena triangularis*, *Zachaeus crista*, and *Leiobunum rotundum*. During our research, species such as *Phalangium opilio*, *Egaenus convexus*, *Nemastoma lugubre*, and *Zachaeus crista* were absent, and additionally, species such as *Lophopilio palpinalis*, *Oligolophus tridens*, *Opilio saxatilis*, and *Lacinius dentiger* were present.

In terms of the geographical distribution, the recorded harvestman species can be classified into four groups (European, Central European, South-Eastern European, Mediterranean) (Stašiov, 2004). *Mitostoma chrysomelas*, *Trogulus tricarinatus*, *Lacinius ephippiatus*, *Leiobunum rotundum*, *Lophopilio palpinalis*, *Oligolophus tridens*, *Opilio saxatilis*, *Rilaena triangularis*, and *Astrobunus laevipes* are European species. *Nemastoma bidentatum sparsum* and *Nelima sempronii* are Central European species. *Opilio canestrinii* is a Mediterranean form invasive in more northern parts of Europe. *Lacinius dentiger* belongs to Central and South-Eastern European species.

The obtained results also indicate the influence of other factors evaluated on harvestman communities. For example, the age of forest stands has a statistically significant impact on the dispersion of harvestman species. Similar findings are also presented by Pekár (2003), who, during a study of spiders and harvestmen in orchards, found that the diversity of observed arachnids was significantly lower in young compared to older plots. This finding was also confirmed by Kataja-aho et al. (2016), who studied the effects of stump harvesting on spiders, ants, harvestmen, ground beetles, and epiedaphic springtails inhabiting boreal Norway spruce forests. Harvestmen were more abundant in the mature forests.

The results of our study provide significant information on the dynamics of harvestman communities in relation to microclimatic variables, environmental characteristics, and management practices within the historical park in Rusovce (Bratislava). Our findings support several of the hypotheses formulated at the beginning of this investigation, particularly regarding the influence of temperature and vegetation structure on the distribution of species of harvestmen. Our first two hypotheses predicted that higher soil temperatures (T1), surface temperatures (T2), and air temperatures (T3) would positively correlate with harvestman abundance and species richness. The RDA results confirmed significant influences of all three temperature measurements on the spatial structure of harvestman species, with *p*-values of 0.02, 0.04, and 0.036 respectively. This supports Hypotheses 1 and 2, indicating that harvestmen are indeed sensitive to microclimatic variations, consistent with findings by Pinto-da-Rocha et al. (2007), who noted that temperature fluctuations can significantly affect the activity and distribution of ectothermic organisms. The temperature can be considered the most important factor significantly influencing the abundance and diversity of harvestmen in our study. We confirmed the significant influence of temperatures measured at different points on the spatial structure of harvestman species (T1–T3). The temperatures had the greatest influence on the species *Leiobunum rotundum*, *Oligolophus tridens*, *Mitostoma chrysomelas*, *Opilio saxatilis*, and *Lacinius ephippiatus*. Several authors confirmed that the structure of harvestman communities is significantly affected by temperature and humidity (Almeida-Neto et al., 2006; Branquart et al., 1995; Gava, 2004; Landsman & Thiel, 2021; Pinto-da-Rocha et al., 2007; Resende et al., 2012; Todd, 1949).

Our analysis also confirmed the significant influence of environmental variables on the dispersion of harvestmen, particularly the number of plant species in the shrub layer (E_2_ species) and the canopy cover of the shrub (E_2_ [%]) and herb layers (E_1_ [%]). The *p*-values for these variables were statistically significant (*p* = 0.025, *p* = 0.011, and *p* = 0.023, respectively), supporting Hypothesis 3. From the results of the analysis, we confirmed that the stand canopy of the tree vegetation layer affects the species *Rilaena triangularis*, *Opilio saxatilis*, *Nelima sempronii*, *Lacinius dentiger*, and *Trogulus tricarinatus* the most. The plant species richness of shrub and tree vegetation layers has the greatest influence on the species *Lacinius ephippiatus*, *Leiobunum rotundum*, *Mitostoma chrysomelas*, and *Oligolophus tridens*. These results align with previous studies, such as those by Bragagnolo et al. (2007), which emphasized the importance of habitat quality (forest structure) and quantity (remnant size) in supporting harvestman communities. During a study of selected groups of arthropods in vineyards in South-Eastern France, Blaise et al. (2022) also point out that plant species richness has a significant positive effect on harvestmen abundances. Liere and Cowal (2024) conducted a survey of selected arthropods in 10 urban community gardens in Seattle, Washington, during the summer of 2019, and confirmed that the species richness of trees and shrubs also increased the abundance of harvestmen. During the examination of harvestmen communities at eight sites within the floodplain forests of three rivers: the Danube in Slovakia, and the Tisa and Begej in Serbia, Litavský et al. (2018) also identified significant positive correlations between the equitability of harvestmen communities and the species richness of plant communities in the shrub layer. Other researchers have also corroborated the influence of tree species on harvestmen communities (Černecká et al., 2017; Rahmani & Mayvan, 2003; Stašiov et al., 1997). For instance, Rahmani and Mayvan (2003) conducted a comparative study on various groups of soil-dwelling invertebrates, including harvestmen, across different forest stands in Neka, northern Iran (Caspian forests). They discovered that the diversity and evenness of the soil communities were greater in the beech forests compared to the hornbeam forests. These authors suggest that the composition of tree species influences the structure of harvestman communities primarily through its impact on microclimatic conditions, particularly temperature and humidity. Stašiov et al. (1997) also validated the impact of tree species composition on the species structure of harvestman communities. In the Malý Polom National Nature Reserve (northern Slovakia), they studied harvestman communities across six locations that varied in the presence of spruce, fir, and beech. They concluded that the composition of tree species in the stands is more significant than terrain exposure and altitude. According to Černecká et al. (2017), the canopy cover of beech forest stands affects the abundance of individual species in epigean assemblages more than their taxonomic composition and the total abundance of captured harvestmen individuals.

Regarding the impact of management measures, we confirm that the presence of invasive plant species had a statistically significant effect on the composition of the species of Opiliones validating Hypothesis 4 (*p* = 0.015). Many authors found that invasive plant species have a significant negative impact on species richness and total abundance of arthropods (Degomez & Wagner, 2001; Elleriis et al., 2015; Litt et al., 2014; Štrobl et al., 2019; van Hengstum et al., 2014). Although many studies highlight the negative impacts of monoculture tree plantations on arthropod diversity (Damptey et al., 2023; Stamps & Linit, 1997; Wang et al., 2019), we recorded the highest species richness of harvestmen within pine monoculture (S7) in our entire study. Our results showed that monoculture tree plantations had a significantly positive effect on the diversity and composition of harvestmen, thus disproving Hypothesis 5 (*p* = 0.02). Interestingly, species such as *Lacinius dentiger*, *Lacinius ephippiatus*, *Opilio saxatilis*, *Rilaena triangularis*, and *Trogulus tricarinatus* preferred linden and pine monocultures, suggesting that some species of harvestmen can adapt to these modified habitats or even thrive in them. This observation is consistent with Litavský et al. (2019b), who recorded the highest diversity of harvestmen in poplar monocultures within the floodplain forests along the Danube near Bratislava.

We did not statistically confirm Hypothesis 6. We did not find significant effects of trampling, mowing, or tree cutting on the overall structure of the Opiliones community structure (*p*-values > 0.5). This may indicate that, while these management practices can alter habitat conditions, their effects on harvestman communities may be less pronounced than those of invasive species and monoculture plantations. This finding suggests that the impact of management practices may vary among species, with some being more resilient to disturbances than others.

Our results indicate the importance of management measures implemented in urban green spaces, influencing the species composition of plants and, consequently, the communities of harvestmen and other arthropods inhabiting similar types of habitats.

Although our study identified significant relationships between harvestman communities and various environmental variables, the complexity of ecological interactions means that other unmeasured factors could also influence species distributions. For example, soil moisture (Almeida-Neto et al., 2006; Branquart et al., 1995; Gava, 2004; Pinto-da-Rocha et al., 2007; Todd, 1949), soil pH, and nutrient availability (Dunger, 1958; Litavský et al., 2018) may play critical roles in shaping harvestman habitats but were not included in the analysis. The omission of these variables could lead to an incomplete understanding of ecological dynamics. Addressing these issues in future research could lead to a more nuanced understanding of the factors influencing harvestman communities and inform effective conservation strategies. Nevertheless, our research represents one of the few recent studies in conservation ecology that specifically examines the environmental impacts on harvestmen communities within urban green spaces. This work fills a critical gap in the literature, providing new insights into how urban environments affect these communities and highlighting the importance of conservation efforts in these environments.

## Conclusions

The results showed that the specific species of harvestmen (e.g., *Leiobunum rotundum*, *Oligolophus tridens*) are significantly influenced by temperature variations and environmental conditions. Harvestmen can be effectively used as bioindicators to monitor ecological changes in urban parks and other anthropogenically influenced ecosystems. Microclimatic conditions, particularly soil and air temperatures, play a critical role in shaping the structure and dynamics of harvestman communities. Understanding the impact of microclimate on harvestmen can inform conservation strategies that maintain or improve habitat quality in urban parks. Plant diversity, cover of vegetation layers, and forest stand age significantly influence the dispersal and composition of harvestmen species. These environmental variables are key to designing management practices that support biodiversity in urban green spaces. Management activities, especially monoculture tree plantations and the presence of invasive species, significantly affect the species composition of harvestmen. Identifying the impact of different management practices can help in developing guidelines for urban park maintenance that enhance biodiversity and ecosystem health. The study identified the species diversity and abundance of harvestmen in different management zones of the park in Rusovce, highlighting areas with higher and lower species richness. Detailed knowledge of species diversity and distribution can guide targeted conservation efforts and prioritize areas for habitat restoration. This finding can be used to refine park management strategies to minimize negative impacts on sensitive species and promote a more diverse and resilient ecosystem.

Our findings emphasize the importance of microclimatic and environmental factors, as well as the effects of human management practices, on the diversity and distribution of harvestmen. This research provides valuable insights into the management of urban parks and the conservation of biodiversity, highlighting the need for informed and sustainable practices to maintain ecological balance in urban environments.

Based on the findings of the study on harvestman communities in the historical park in Rusovce, several recommendations can be made for the management of urban parks and other green spaces:

- Increase plant diversity and structural complexity in the park by incorporating a mix of native plant species across different vegetation layers (tree, shrub, and herbaceous layers). This can be achieved by implementing a planting strategy that supports a diverse range of flora, which can provide essential microhabitats for harvestmen and other wildlife
- Adopt sustainable management practices that minimize ecosystem disruption in the park, with a particular focus on controlling invasive species and managing monocultural plantings
- Establish a program to monitor microclimatic variables (e.g., soil and air temperature and humidity) to better understand their impact on local biodiversity. This information can help inform vegetation management decisions and habitat restoration efforts, ensuring that conditions remain favorable for sensitive species
- Conducting ecological research within urban green areas and adapting management measures for the given area based on new findings

## Data Availability

No datasets were generated or analysed during the current study.
